# Occurrence and Distribution of 13 Trichothecene Toxins in Naturally Contaminated Maize Plants in Germany

**DOI:** 10.3390/toxins4100778

**Published:** 2012-09-28

**Authors:** Margit Schollenberger, Hans-Martin Müller, Katrin Ernst, Sarah Sondermann, Melanie Liebscher, Claudia Schlecker, Gerald Wischer, Winfried Drochner, Karin Hartung, Hans-Peter Piepho

**Affiliations:** 1 Institute of Animal Nutrition, University of Hohenheim, Emil-Wolff-Str. 10, 70599 Stuttgart, Germany; Email: hans-martin.mueller@t-online.de (H.-M.M.); katrin.ernst@biomin.net (K.E.); Sarah.Sondermann@deutsche-tiernahrung.de (S.S.); m.liebscher@uni-hohenheim.de (M.L.); inst450@uni-hohenheim.de (C.S.); gerald.wischer@googlemail.com (G.W.); winfried.drochner@uni-hohenheim.de (W.D.); 2 Bioinformatics Unit, Institute of Crop Science, University of Hohenheim, Fruwirthstraße 23, 70599 Stuttgart, Germany; Email: karin.hartung@uni-hohenheim.de (K.H.); piepho@uni-hohenheim.de (H.-P.P.)

**Keywords:** A-type and B-type trichothecenes, contamination pattern, distribution, maize, multitrichothecene contamination, fractions

## Abstract

The objective of the present study was to monitor the occurrence and distribution of a spectrum of trichothecene toxins in different parts of maize plants. Therefore maize plants were sampled randomly from 13 fields in southwest Germany and the fractions kernels, cobs, husks, stalks, leaves and rudimentary ears were analyzed for eight A-type and five B-type trichothecenes. Each of the toxins was found in at least three of the total of 78 samples. The study revealed that both A-type and B-type trichothecenes may be present in all parts of the maize plant but may be unevenly distributed. For the contents of deoxynivalenol, 3- and 15-acetyldeoxynivalenol, nivalenol, scirpentriol, 15-monoacetoxyscirpenol, HT-2 and T-2 toxin significant differences (*p* < 0.05) were found between different parts of the maize plants whereas no significant differences were observed for fusarenon-X, 4,15-diacetoxyscirpenol, neosolaniol, T-2 triol and T-2 tetraol. Up to twelve toxins co-occurring in one sample were detected. As a group B-type trichothecenes dominated over A-type trichothecenes concerning incidences and levels. Contamination was strongest with rudimentary ears based on incidence and mean and maximum contents; mean contents with few exceptions tended towards a higher level than in other fractions with significant (*p* < 0.05) differences compared to leaves for seven toxins.

## 1. Introduction

Maize (*Zea mays *L.) worldwide is an important cereal crop grown over a variety of climatic conditions. The kernel serves as raw material for foods and food components as well as for feed. Whole plants or plant parts are used as feedstuff such as forage or corn cob mix. 

Maize plants are susceptible to *Fusarium* infestation and therefore together with deriving feedstuffs may contain trichothecene toxins of the A-type as well as of the B-type [1,2,3,4,5]. There are several routes by which the fungus gains entry into the plant. Diseases resulting from *Fusarium* infection are seed rot, seedling blight, root rot, stalk rot, and ear rot. Primary infection of the stalk can be caused by fungi overwintered in stubbles on the fields [6], and a secondary ear rot infection may start together with flowering. Species usually isolated from stalk rot of maize in Europe are *F. graminearum*, *F. culmorum* and *F. verticillioides* [7]. Spore entry into maize ears can occur through wounds or by growth of mycelium along the silk down to the kernel and cob from spores germinating on the silk [8]. The phase of maize contamination with *Fusarium* with the greatest mycotoxicological concern is ear rot, but the formation of mycotoxins in rotted stalks, infected leaves and in whole plants could also represent a significant risk for forage and silo maize [9].

Although there is much information about the incidence of *Fusarium* infection and *Fusarium* toxins in maize kernels, little is known on their incidence and distribution in the maize plant itself. 

Trichothecenes were found in whole plants of maize or silage by several authors [2,10,11,12]. Ten different trichothecenes of the A-type as well as of the B-type were detected in whole maize plants by Schollenberger *et al.* [3].

A limited number of studies have dealt with the distribution of trichothecene toxins in different parts of the maize plant after natural [13,14] or artificial inoculation [15,16] with *Fusaria*. All these studies were restricted to B-type trichothecenes, mainly deoxynivalenol (DON), in part also nivalenol (NIV) [13,14] and 15-acetyldeoxynivalenol (15-ADON) [16]. However, inoculation studies generally may not truly reflect natural events [14]. Furthermore, for the assessment of risks posed by *Fusarium* infection of maize plants to animal and human health it seems to be essential to determine a broad spectrum of trichothecenes including also toxins of the A-type. In this context it is of interest that trichothecenes of the latter type are much more toxic than type-B trichothecenes [17]. To our knowledge no data exist about the distribution of A-type trichothecenes in maize plants. As whole plants or different parts of the plants are used as feedstuff, the toxin content of fractions of the plant other than the kernel is significant to animal growth and health [13]. Therefore in the present study a spectrum of 13 A-type and B-type trichothecenes was analyzed in different parts of maize plants. 

The fractions examined were kernel, cob and husk from the ears, rudimentary ears, stalks and leaves. The toxins analyzed were the A-type trichothecenes scirpentriol (SCIRP), 15-monoacetoxyscirpenol (MAS), 4,15-diacetoxyscirpenol (DAS), T-2 and HT-2 toxins (T-2, HT-2), T-2 triol (T-2,3), T-2 tetraol (T-2,4) and neosoloaniol (NEO), and the B-type trichothecenes DON, 3- acetyldeoxynivalenol (3-ADON), 15-ADON, NIV and fusarenone-X (FUS-X). 

## 2. Results

Six maize fractions with 13 samples per fraction were analyzed for the B-type trichothecenes, DON, 3-and 15- ADON, NIV, FUS-X, and the A-type trichothecenes, SCIRP, MAS, DAS, NEO, HT-2, T-2, T-2,3, and T-2,4. All of these toxins were found in at least three of the total of 78 samples. 

The spectrum of toxins in each fraction, their incidences and mean and maximum contents are described in Table 1 and Table 2. Mean contents were based on the total of 13 samples analyzed per fraction, with the content of negative samples assumed at half the detection limit.

Contamination varied with toxin and fraction. Thus DON, 3-ADON, 15-ADON, HT-2, and T-2 were found in all six fractions, NIV in five, FUS-X, SCIRP and MAS in four, T-2,4 in three, T-2,3 and NEO in two, and DAS in one fraction, with the incidences per fraction varying between 8% and 100%. 

For the fractions rudimentary ears, kernels, cobs, husks, stalks and leaves the averages of co-occurring toxins were 8, 3, 3, 5, 2, and 1, and the maximum numbers of co-occurring toxins were 12, 9, 7, 8, 5, and 3, respectively. 

For the different toxins maximum values were not generally issued from the same sample. Thus in each of the six fractions analyzed the highest contamination of the 13 toxins was found in four to five different samples. Furthermore not all fractions of one field showed maximum contents for a specific toxin. Thus in the six fractions analyzed the highest contents of DON, 15-ADON, 3-ADON, HT-2 and T-2 were found in five–six different samples showing that the maximum contamination was not necessarily found in the whole plant but only in parts of it. 

For the toxins DON, 3-ADON, 15-ADON, NIV, SCIRP, MAS, HT-2 and T-2 significant differences (*p* < 0.05) were found for toxin contents between some fractions whereas no significant differences were observed for FUS-X, DAS, NEO, T-2,3 and T-2,4. The contamination with few exceptions was strongest for rudimentary ears based on incidence, mean and maximum contents. Thus each of the toxins analyzed was detected in rudimentary ears, including DAS, which occurred exclusively here. The NIV contents with a mean at 27,209 µg/kg and a maximum at 125,325 µg/kg were the highest compared to all other toxins and fractions. Incidences of SCIRP, MAS and DAS were at 92%, 85% and 31% and maximum amounts were at 45,273, 3344 and 1294 µg/kg. Markedly high contents in this fraction were also found for DON, 3-ADON, and T-2,4, with a mean at 2762–18,071 µg/kg, and a maximum at 32,293–88,907 µg/kg. The mean contents of NIV, SCIRP and MAS were significantly (*p* < 0.05) higher in rudimentary ears compared to all other fractions; the contents of DON, 3-ADON, 15-ADON were significantly different between rudimentary ears and four other fractions. 

In leaves only five toxins were detected, the mean and maximum contents were below 100 µg/kg with the exception of the maximum value of DON of 323 µg/kg. 

In cobs the mean contents of 15-ADON, DON and NIV ranged between 4415 and 9182 µg/kg, and maximum contents were between 19,106 and 105,217 µg/kg. 

Within the ear deriving fractions kernels, cobs and husks no significant differences in toxin contents were found with the exceptions of 15-ADON, which was significantly higher in cobs and MAS, which was higher in husk compared to the other two fractions. In the three ear deriving fractions the mean contents of DON and 15-ADON were significantly higher than in leaves. No significant differences were found between leaves and stalks.

As can be seen from Table 1 and Table 2 differences in the degree of contamination were found between A- and B-type trichothecenes analyzed with the group of B-type trichothecenes dominating over A-type trichothecenes concerning both incidences and levels. In the total of 78 samples the percentage of positive samples was at 64%, 36%, 60%, 37%, 6% for the B-type-trichothecenes DON, 3-and 15- ADON, NIV, FUS-X, and at 27%, 27%, 21%, 5%, 53%, 37%, 4%, 12% for the A-type trichothecenes SCIRP, MAS, DAS, NEO, HT-2, T-2, T-2,3, and T-2,4, respectively. Mean contents were at 4326, 740, 963, 7078 and 58 µg/kg for the B-type and 1984, 173, 33, 10, 175, 38, 14 and 476 µg/kg for the A-type trichothecenes with the content of negative samples assumed at half the detection limit. 

## 3. Discussion

The results presented show that at least five B-type and eight A-type trichothecenes may occur in maize plants. This multitrichothecene contamination is consistent with findings of authors who examined whole plant material or silages [3,12]. Schollenberger *et al.* [3] (2006) analyzed a total of 13 trichothecenes amongst others in maize plants and maize silage harvested in southwest Germany, and found four out of five B-type trichothecenes (DON, 3- and 15 ADON, NIV) and six out of eight A-type trichothecenes (HT-2, T-2, T-2,3, T-2,4, SCIRP, MAS) in maize plants, as well as DON, 15-ADON, NIV, HT-2, SCIRP and MAS in maize silage. 

The co-occurrence of toxins is of interest amongst others for the spectrum of producing fungi and for their toxic capacity*.* Generally, producers may occur simultaneously or in quick succession [18]. According to Bottalico [7], epidemics induced by *F. graminearum* and *F. culmorum*, are usually responsible for the occurrence in maize ears of DON and 3-ADON; NIV and FUS-X could be produced by NIV chemotypes of *F. graminearum *and or *F. crookwellense.* Epidemics of *F. sporotrichioides* usually led to the accumulation of T-2 derivatives in infected ears, epidemics of *F. poae* were responsible not only for the accumulation of DAS and MAS, but also of NIV and FUS-X. In this context it should be considered that the occurrence and prevalence of *Fusarium* species varies from region to region, and year to year, depending on climatic conditions (temperature and rain) and farming practices, including tillage, crop rotation, fertilization and planting area [9]. Dorn *et al.* [19] additionally reviewed the influence of harvesting time, interactions between maize hybrids and environment, nutrient state of the plant, infestation by arthropods on *Fusarium* species composition as well as mycotoxin concentrations. 

Differences were observed in the frequency of occurrence of the particular toxins. Whereas DON was found in at least five samples of each fraction some toxins such as NIV are detected in a minority of samples with very high levels thus elevating the mean toxin concentration of a fraction considerably. This was observed for example for NIV in the fractions corn and cobs. 

According to the present study both A-type and B-type trichothecenes may be present in all parts of the maize plant but may be unevenly distributed. Thus a very high contamination was observed for rudimentary ears concerning number and levels of trichothecenes (Table 1 and Table 2). This is consistent with findings of Oldenburg *et al.* [15], who reported very high DON concentrations in rudimentary ears after artificial infection of the plants. These authors observed that the infection and subsequent contamination of the rudimentary ears obviously proceeded independently from stalk infection, similar to maize kernel infection, which was not correlated with an infection of the roots. Mean and maximum contents of MAS and SCIRP detected in rudimentary ears (Table 2) are considerably higher than found previously in kernel, plant and silage, with maximum contents in plants of up to 916 µg/kg for SCIRP, and 85 µg/kg for MAS [3]. Likewise, contents of the two toxins in other substrates of plant origin were within a markedly lower range compared to rudimentary ears, except for banana fruit from India [20]. Though the share of rudimentary ears in whole maize plants is limited, their outstanding high toxin content found in the present study may contribute significantly to the trichothecene contamination of maize silage. Therefore a reduction of rudimentary ears by plant breeding measures might be favourable to lower the trichothecene content of this feedstuff.

**Table 1:** B-type trichothecene toxins in six fractions of maize plants. Thirteen samples were analyzed per fraction. Toxin contents in microgram per kilogram dry matter.

Fraction		DON	3-ADON	15-ADON	NIV	FUS-X
Kernels	n pos ^1^	7	4	8	3	2
	Mean ^2^	809 ^b^	24 ^a^	114 ^b^	3,569 ^ab^	29 ^a^
	Max	3,118	91	596	43,064	227
Cobs	n pos	8	5	9	4	1
	Mean	6,486 ^bc^	645 ^ab^	4,415 ^c^	9,182 ^ab^	243 ^a^
	Max	31,828	6,048	19,106	105,217	3,033
Husks	n pos	11	3	9	7	1
	Mean	363 ^b^	44 ^a^	178 ^b^	2,407 ^b^	34 ^a^
	Max	1,227	361	978	10,079	309
Rud. Ears3	n pos	13	13	13	12	1
	Mean	18,071 ^c^	3,668 ^b^	846 ^c^	27,209 ^c^	18 ^a^
	Max	88,907	32,293	4,952	125,325	105
Stalks	n pos	5	2	6	3	0
	Mean	143 ^a^	45 ^a^	215 ^ab^	95 ^ab^	11 ^a^
	Max	709	430	1.525	928	11
Leaves	n pos	6	1	2	0	0
	Mean	85 ^a^	12 ^a^	10 ^a^	7 ^a^	11 ^a^
	Max	323	32	72	7	11

^1^ Number of positive samples; ^2^ Mean and maximum content (µg/kg), with the content of negative samples set at half the detection limit; ^3^ Rudimentary ears; Mean contents with different superscripts within one column are significantly different (*p* < 0.05).

**Table 2:** A-type trichothecene toxins in six fractions of maize plants. Thirteen samples were analyzed per fraction. Toxin contents in microgram per kilogramdry matter.

Fraction		SCIRP	MAS	DAS	NEO	T-2,3	T-2,4	HT-2	T-2
Kernels	n pos ^1^	2	1	0	1	1	1	4	3
	Mean ^2^	52^a^	8 ^a^	7 ^a^	18^a^	8 ^a^	26 ^a^	38 ^ab^	36 ^ab^
	Max ^2^	343	30	7	178	24	170	365	429
Cob	n pos	0	0	0	0	0	0	5	4
	Mean	8 ^a^	6 ^a^	7 ^a^	5 ^a^	7 ^a^	14 ^a^	76 ^ab^	13 ^ab^
	Max	8	6	7	5	7	14	709	61
Husks	n pos.	5	6	0	0	0	1	12	10
	Mean	265 ^a^	24 ^b^	7 ^a^	5 ^a^	7 ^a^	27 ^a^	114 ^b^	28 ^b^
	Max	1,095	94	7	5	7	179	595	170
Rudimentary ears	n pos	12	11	4	3	2	4	11	6
	Mean	11,549 ^b^	982 ^c^	162 ^a^	22 ^a^	49 ^a^	2,762 ^a^	777 ^b^	127 ^ab^
	Max	45,273	3,344	1,294	143	506	32,792	7,792	926
Stalks	n pos	2	3	0	0	0	0	6	4
	Mean	20 ^a^	13 ^ab^	7 ^a^	5 ^a^	7 ^a^	14 ^a^	87 ^a^	21 ^a^
	Max	95	48	7	5	7	14	987	135
Leaves	n pos	0	0	0	0	0	0	3	2
	Mean	8 ^a^	6 ^a^	7 ^a^	5 ^a^	7 ^a^	14 ^a^	7 ^a^	5 ^a^
	Max	8	6	7	5	7	14	24	26

^1^ Number of positive samples; ^2^ Mean and maximum content (µg/kg), with the content of negative samples set at half the detection limit. Mean contents with different superscripts within one column are significantly different (*p* < 0.05).

High levels of B-type trichothecenes in the cob may especially be of concern when this fraction is ground for corn cob mix or ensiled corn cob meal and used in pig nutrition. 

An uneven distribution of DON, acetylDONs and NIV in different parts of the maize plant has been described by several authors. Thus DON and NIV occurred at higher concentration in cobs than in kernels [8,13,14,21]. According to Perkowski *et al.* [22] the concentration of DON, 3- and 15- ADON on average was higher in stalks of maize plants compared to kernels. Data about the distribution of A-type trichothecenes in maize plants to our knowledge are not available. 

Different toxin spectra and varying extent of contamination of plant organs (Table 1 and Table 2) may result amongst others from different *Fusarium* populations on these fractions. Whereas Eckard *et al.* [12] did not observe any infection specificity for certain plant parts, several authors reported an uneven distribution of *Fusarium* species between maize stalk and ear [23,24,25] and within the ear deriving fractions [14]. There are several routes by which *Fusaria* gain entry into the plant including wounds by insects and birds, seed transmission and potentially root colonization. Distribution of toxins may vary according to the prevalent way of plant infection. In the present study, ear derived fractions were comparably highly contaminated whereas leaf and stalk fraction showed lower trichothecene concentrations. However, DiMenna *et al.* [13] reported that kernels may be one of the least contaminated parts of the maize plant. When interpreting toxin levels in stalk fractions it must be taken into consideration that presumably in most cases rudimentary ears are not separated from stalk but analyzed together with this fraction and thus may markedly influence the result of toxin analysis. 

The trichothecene pattern of different maize organs may depend not only on the *Fusarium* species present but also on the effect of multiple factors affecting their toxin production, including temperature, growth medium and water potential. The optimum climatic conditions for mycotoxin production in infected grains can vary markedly among *Fusarium* species and even among isolates within one species. Most studies indicate that high moisture and warm temperatures favour production of all classes of mycotoxins [18].

The possibility of translocation of toxins within the plant should also be taken into consideration. Thus a translocation of DON within the maize plant was assumed by several authors [8,16,26]. Oldenburg *et al.* [15] however artificially inoculated maize plants using *Fusarium graminearum*-inoculated oat kernels spread on the soil surface of the experimental field. The authors found a close relation between *Fusarium* biomass and DON concentrations determined in different maize organs and therefore concluded that the mycotoxin was not translocated within the plant but primarily remained in infected organs, where it was produced by the fungus. 

In the present study a predominance of B-type trichothecenes compared to A-type trichothecenes concerning incidence and level was found for several fractions analyzed but in this context the different toxicity of these substances should be taken into consideration. Thus the Scientific Committee on Food [27] published acceptable daily intakes (ADI) for the B-type trichothecenes NIV and DON of 0.7 and 1 µg/kg bodyweight, and for the sum of the A-type trichothecenes HT-2 and T-2 of 0.06 µg/kg bodyweight. Recently the group ADI for HT-2 and T-2 was changed to 0.1 µg/kg bodyweight [28].

The scirpentriol toxins DAS, MAS, SCIRP, amongst others, appeared to be at least as potent as the better studied T-2 toxin [29]. 

With regard to the toxicity of maize fractions it should be considered that in plants chopped for silage making, uncontaminated fractions may be mixed with those strongly contaminated with mycotoxins [13], and that trichothecenes in naturally contaminated corn cob mixes and corn silages are still present after ensiling [3,13]. Due to the unequal distribution of toxins in the maize plant, toxic effects on animals will vary according to the fractions offered for consumption. The biological reactions following ingestion of one or a combination of mycotoxins may vary from acute disease to economical losses through clinically obscure changes in growth, production and immunosuppression [30]. The co-occurrence of several mycotoxins with specific chemical traits and modes of action can be regarded as a serious problem because of possible additive and/or synergistic effects [9]. 

## 4. Materials and Methods

### 4.1. Samples

Collection of samples was between mid-September and mid-October 2008 prior to harvest in 13 different locations in Southwest Germany. Ten randomly selected plants per field were cut at ground level and transported to the laboratory. Each plant was dissected into six fractions comprising kernels, cobs from which the kernels had been removed, husks, rudimentary ears, stalk, and leaves. The corresponding fractions from ten plants per field were pooled into one sample, yielding 13 samples per fraction and a total of 78 samples for six fractions. For the fractions stalks and leaves, the plant segments were chopped before mixing, split, and a subsample of approximately 500 g was dried. Samples were dried at 40 °C, ground to pass through a 1 mm sieve and stored at −20 °C prior to analysis for a spectrum of 13 trichothecene toxins. 

### 4.2. Toxin Analysis

Mycotoxin standards were bought at Sigma (Deisenhofen, Germany). Analysis was carried out as described in detail previously [25,31]. In brief, extraction was performed with a mixture of acetonitrile and water followed by liquid/liquid extraction with hexane. Clean up was carried out by solid phase extraction using a florisil (Varian, Mega Bond Elut Florisil, 5 g/20 mL) and a cation exchange cartridge (Varian, Megabond Elut CBA, 1 g/6 mL). For elution from a cation exchange cartridge a mixture of acetic acid/methanol (60/40, *v*/*v*) was used. Derivatization was done with trifluoroacetic anhydride, using verrucarol to ensure derivatization efficiency. Separation and quantitation was performed by GC-MS using a Magnum Ion Trap system in the chemical ionisation mode with isobutane as reactant gas. Detection limits were assessed at a signal to noise ratio of 3:1 and were 16, 12, 14, 10, 14, 28, 6, 4, 6, 8, 20, 14 and 22 µg/kg for SCIRP, MAS, DAS, NEO, T-2 triol, T-2 tetraol, HT-2, T-2, DON, 15-ADON, 3-ADON, NIV and FUS-X, respectively. Quantitation limits were at a signal to noise ratio of 6:1. Toxin contents between detection and quantification limit were calculated as the average. Recovery rates were determined using a spiking level of 1000 µg/kg for the toxins NIV, DON, FUS-X, 15-ADON, 3-ADON, HT-2 and T-2 in leaves and stem. Mean recovery rates were between 59% and 115% with the exception of NIV and DON in stem with recovery rates of 41% and 42%, respectively. Results were not corrected for recovery. Naturally contaminated in-house reference material was regularly analyzed to provide for accuracy of results. Toxin content was based on dry matter which was determined according to Naumann and Basler [32].

### 4.3. Statistics

The Wilkoxon signed-rank test was used for examination of statistical significance of differences in medium toxin contents between fractions and was performed by the procedure “univariate“of SAS (SAS Institute Inc., release 2002–2003, (*r*), 9.1). 

## 5. Conclusion

This is the first time that the distribution not only of selected trichothecenes but of a total of five B-type and in addition eight A-type trichothecenes has been reported in different parts of the maize plant. The study revealed the possibility of a multitrichothecene contamination and an uneven distribution of these toxins in different fractions, with elevated toxin contamination of rudimentary ears compared to other fractions. 
